# Bacterial Communities Vary from Different Scleractinian Coral Species and between Bleached and Non-Bleached Corals

**DOI:** 10.1128/spectrum.04910-22

**Published:** 2023-05-16

**Authors:** Meiting Xu, Keke Cheng, Baohua Xiao, Mengmeng Tong, Zhonghua Cai, Mui-Choo Jong, Guofu Chen, Jin Zhou

**Affiliations:** a School of Environment, Harbin Institute of Technology, Harbin, People’s Republic of China; b School of Marine Science and Technology, Harbin Institute of Technology (Weihai), Weihai, Shandong Province, People’s Republic of China; c Shenzhen Public Platform for Screening and Application of Marine Microbial Resources, Institute for Ocean Engineering, Shenzhen International Graduate School, Tsinghua University, Shenzhen, People’s Republic of China; d Shenzhen Institute of Guangdong Ocean University, Shenzhen, People’s Republic of China; e Ocean College, Zhejiang University, Zhoushan, People’s Republic of China; f Institute of Environment and Ecology, Shenzhen International Graduate School, Tsinghua University, Shenzhen, People’s Republic of China; Nanjing Institute of Geography and Limnology Chinese Academy of Sciences

**Keywords:** coral health status, bleaching, bacterial communities, network relationship, host-dependent profile

## Abstract

Bleaching is one of the most relevant factors implicated in the integrity of coral reef ecosystems, with the increasing frequency and intensity of damaging events representing a serious threat to reef biodiversity. Here, we analyzed changes in coral-associated bacteria from three types of non-bleached and bleached scleractinian corals (Acropora digitifera, Galaxea fascicularis, and Porites pukoensis) in Hainan Luhuitou peninsula coastal areas. The community structure of symbiotic bacteria differed significantly among the three apparently healthy corals. The bleached corals had higher bacterial alpha diversity and some specific bacteria genera, including *Ruegeria*, *Methyloceanibacter*, *Filomicrobium*, *Halioglobus*, *Rubripirellula*, *Rhodopirellula*, *Silicimonas*, *Blastopirellula*, Sva0996 marine group, *Woeseia*, and unclassified_c_Gammaproteobacteria, were consistently increased in bleached groups. Network analysis revealed significantly different degrees of modularity between bleached and non-bleached groups at the bacterial genus level, and a higher proportion of links was dominated by positive co-occurrences. Functional prediction analysis illustrated that coral-associated bacteria remained relatively consistent in the bleached and non-bleached groups. Structure equation modeling revealed that the bacterial community diversity and function were directly influenced by host and environment factors. These findings suggested that coral-associated bacterial responses to bleaching occur in a host-dependent manner, informing novel strategies for restoring coral and aiding adaption to bleaching stress.

**IMPORTANCE** Accumulating evidence indicates that coral-associated bacteria play an important role in the health of holobionts. However, the variability of the symbiotic bacterial community structure among coral species with different coral health statuses remains largely unknown. Here, we investigated three apparent non-bleached (healthy) and bleached coral species (sampled *in situ*), involving related symbiotic bacterial profiles, including composition, alpha diversity, network relationship, and potential function. Structural equation modeling analysis was used to analyze the relationship between coral status and abiotic and biotic factors. The bacterial community structure of different groups was shown to exhibit host-specific traits. Both host and environmental impacts had primary effects on coral-associated microbial communities. Future studies are needed to identify the mechanisms that mediate divergent microbial consortia.

## INTRODUCTION

The coral holobiont harbors a diverse assemblage of microorganisms (1). Taking the symbiotic microalgae as an example, impressive progress has revealed endosymbiotic dinoflagellates functional roles and photosynthate mechanisms, which transfer the majority of the coral’s energy requirements (2, 3), promoting calcification rates and coral growth, and in return, the hosts supply an acidic microenvironment and inorganic nutrients for the symbiotic dinoflagellates (4–6). Meanwhile, microbial symbionts, such as bacteria, facilitate host nutrient cycling and support more protective gene functions (7). Symbiotic partnerships, which provide niches for a wide range of diverse coral reef organisms, are a primary source of structure and nutrition in oceans.

The health of the coral holobiont hinges on multiple external and internal factors. The former includes temperature changes (8), eutrophication (9, 10), ocean acidification (11), and other anthropogenic environmental stresses (such as plastic pollution and overfishing) (12). All of these factors can destabilize the symbiosis between the host and their symbionts, leading to massive mortality and bleaching events (13). The latter emphasizes that the potential contributions of the mutualistic symbiosis among coral symbionts, members of the host, dinoflagellate symbiont *Symbiodinum*, and microorganisms are essential to host-symbiont homeostasis and integrity (14). However, the disruption of the symbiotic relationship can lead to the destruction of coral tissue and the escape of zooxanthellae symbionts from coral hosts, resulting in bleaching (15, 16). Moreover, extensive research into coral-associated bacteria has increased attention (17–20). Coral-associated bacteria play a role in coral health (21, 22). Different putative or opportunistic pathogens have been identified and are shown to cause coral disease and bleaching (18, 23–25). Independent experimental studies have also defined coral bleaching as a bacterial dysbiosis within the coral holobiont (26).

Nonetheless, regarding potential adaptation to stress (27), studies have indicated that the bleaching tolerance of some corals explains their ability to resist coral bleaching and substantially increases their survival (28, 29). Bleaching tolerance is a location-and species-specific trait, which is associated with the composition of the bacterial community (29, 30). There is also increasing evidence to suggest that the microbiome can regulate host resistance to thermal-induced bleaching (31, 32). Nonetheless, comparatively little is known about the structural and functional characteristics of symbiotic bacterial communities with different coral health statuses. Comparative studies on shifts in the microbial community in individuals of different coral species with non-bleached and bleached are also lacking.

Subsequently, to comprehensively address how coral bacteria differ by coral type and health status, we collected non-bleached and bleached coral colony fragments from one reef site (Hainan Luhuitou) to ensure the same level of environmental stress. We hypothesized that the bacterial community structure would differ among the different coral types and that bleached individuals would also exhibit obvious changes. To test this, three apparent non-bleached (healthy) and bleached coral species (Acropora digitifera, Galaxea fascicularis, and Porites pukoensis) were sampled *in situ*, and the related symbiotic bacterial profiles were investigated using the 16S rRNA gene pyrosequencing, including the composition, biodiversity, network relationship, as well as the potential function. This work aims to provide a basis for understanding the ecological relationships between corals and their symbiotic communities under bleached and non-bleached. Meanwhile, the knowledge gained from this study may pave the way for new therapeutic approaches and bioremediation techniques to combat coral bleaching.

## RESULTS

### Overall taxonomic characteristics and alpha diversity.

In this study, we applied high-throughput 16S rRNA gene sequencing methods to simultaneously assess the diversity of prokaryotic microbial communities from the two phenotypes (non-bleached and bleached) of three species of stony corals and seawater. In total, 2,563,874 16S rRNA sequences were recorded after quality control and sequence filtering, which were clustered into 13,357 operational taxonomic units (OTUs) at 97% similarity level, respectively. Before alpha and beta analysis and to equalize sequencing depth, OTU tables were rarefied to a minimum number per sample (22,282 quality sequences per bacterial sample).

**FIG 1:**
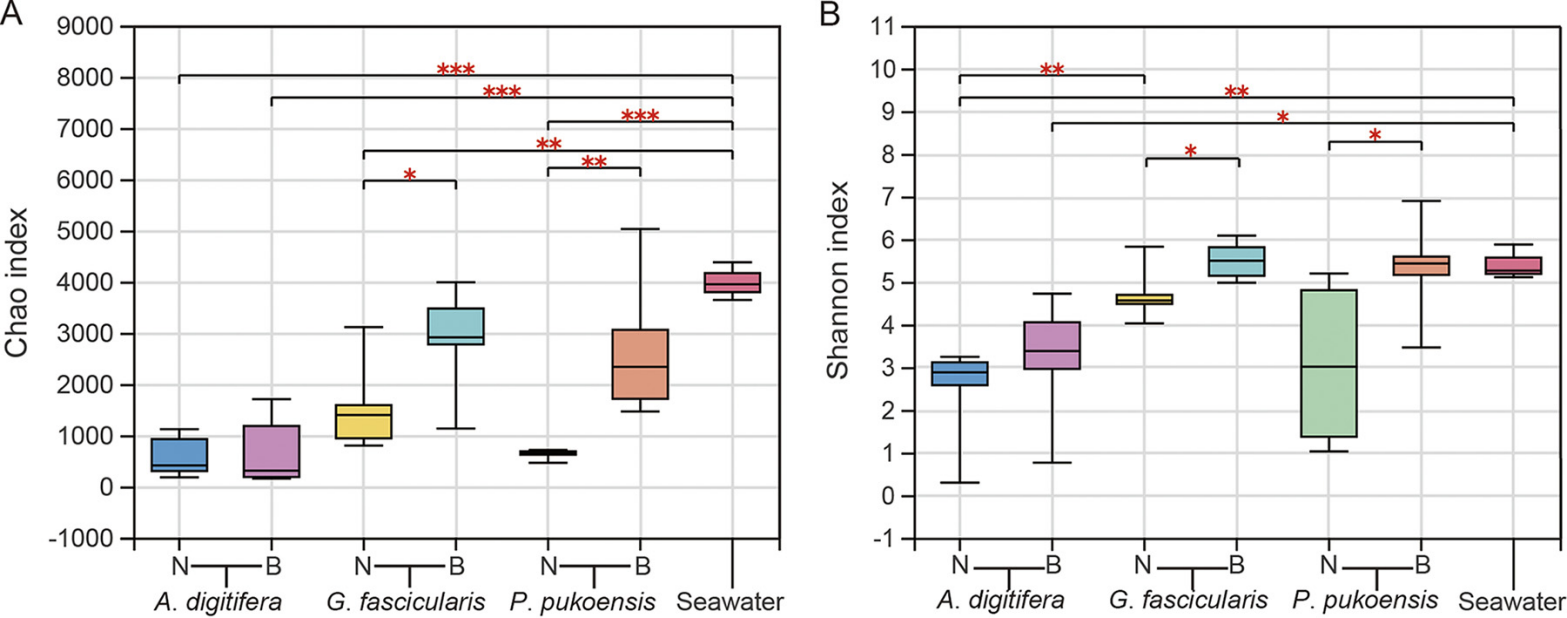
Compassion of alpha diversity indices based on 16S rRNA genes sequencing (A, B). Asterisks indicate significant differences (*, *P ≤ *0.05; **, *P ≤ *0.01; ***, *P ≤ *0.001) based on the Student's *t* test. N, non-bleached corals; B, bleached corals.

In the taxonomically assigned OTUs from the prokaryotic data set, the Good’s coverage values ranged from 99.28% to 99.99%, revealing that sequencing depth was sufficient to capture the majority of the bacterial community (see Table S1 in the supplemental material). Principle seawater had higher alpha diversity for bacteria than coral samples. The Shannon and Chao1 indices, respectively, represent the diversity and richness of communities, and the OTU richness (Chao 1) of seawater was highest across all samples. In the coral samples, bleached coral species were significantly higher in richness and evenness (Chao 1) compared to non-bleached, except for *A*. *digitifera* (Fig. 1A). The same difference was observed regarding Shannon indices with increased diversity in bleached *A*. *digitifera*, *G*. *fascicularis*, and *P*. *pukoensis* (Fig. 1B; Table S1). This shows that bleached *G*. *fascicularis* and *P*. *pukoensis* samples possessed diversity that was more approximate to seawater samples (Fig. 1B). A Student’s *t* test also showed differences in alpha diversity between the three coral species under non-bleached and bleached conditions (shown in Table S2).

### Prokaryotic community compositions and features.

**FIG 2:**
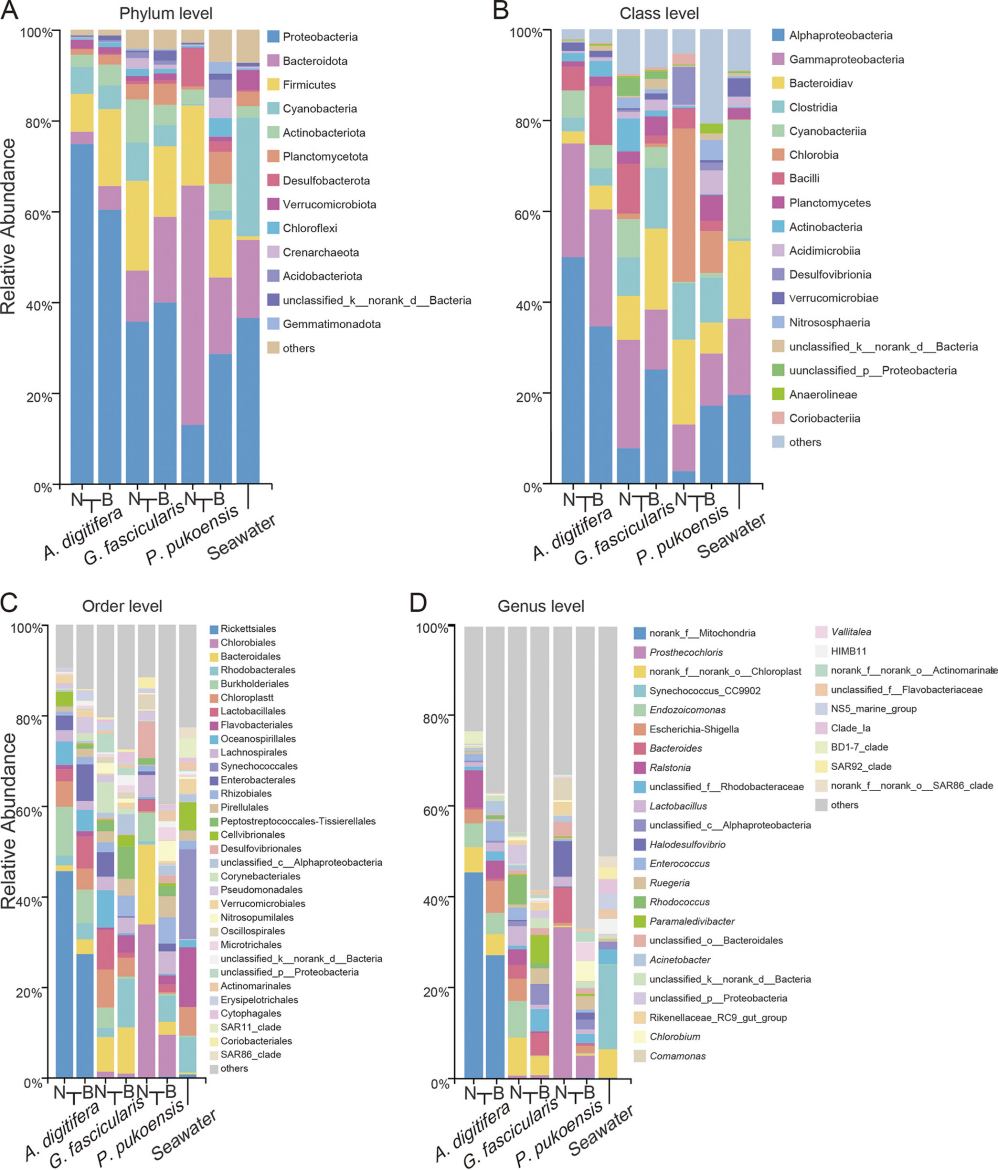
Composition of microbial prokaryotes present in three non-bleached (N) and bleached (B) coral species and seawater. Bar plots show the taxonomic classification of OTU in each sample at the phyla (A), class (B), order (C), and genus (D) levels (other: relative abundance <2%).

The taxonomically assigned OTUs from all samples involved 384 archaeal OTUs and 11,655 bacterial OTUs after the removal of unclassified at the domain, belonged to 66 phyla, 183 classes, 456 orders, 790 families, 1,644 genera, and 3,283 species. The relative abundance at the phylum level presented in Fig. 2A revealed that the dominant phylum was *Proteobacteria* (near 12.9 to 74.8%) in the coral and seawater groups but not in the non-bleached *P*. *pukoensis* (*Bacteroidota* was the most dominant phylum at 52.7% average relative abundance). After *Proteobacteria* and *Bacteroidota* were the predominant phylums, followed by *Firmicutes*, *Cyanobacteria*, and *Actinobacteriota*. Among them, *Fimicutes* showed a relative abundance ranging from 8.3% to 19.7% among the coral species and an abundance of only 0.7% in seawater. Unlike in Fimicutes, *Cyanobateria* showed a different distribution pattern in coral and seawater. *Cyanobateria* was dominant (26.2%) in seawater with only 0.3% to 8.4% in coral samples. *Actinobacteriota* did not exhibit significant differences in the relative abundance in all tested samples, which ranged from 2.4 to 9.4%.

At the class level (Fig. 2B), there were remarkable differences between the seawater and coral samples. While the former was dominated by *Cyanobacteria* (26.1%) and *Alphaproteobacteria* (19.4%), the latter was dominated by *Gammaproteobacteria* (16.7%) and *Bacteriodia* (17.1%). The most obvious change in bleached *A*. *digitifera* was increased *Bacilli* and *Actinobacteria*. The dominant class also changed markedly in *G*. *fascicularis* and *P*. *pukoensis*. For *G*. *fascicularis*, the dominant class in non-bleached coral was *Gammaproteobacteria* (23.8%) and A*lphaproteobacteria* (25.0%) in bleached coral. Furthermore, additional obvious changes in bleached coral were a reduction in *Bacilli* and *Actinobacteria*. For *P*. *pukoensis*, the *Chlorobia* and *Bacteroidiav* were decreased by more than 3-fold and *Alphaproteobacteria* increased almost 6-fold compared with the non-bleached samples.

At the order level, the differences between species were further amplified. The relative abundance of the three analyzed coral species fluctuated by coral type (Fig. 2C). The dominant order-species in the healthy *A*. *digitifera*, *G*. *fascicularis*, and *P*. *pukoensis* were *Rickettsiales*, *Chloroplast*, and *Chlorobiales*, respectively. In bleached conditions, these members were reduced in relative abundance including *Burkholderiales* and *Chloroplast* (Fig. 2C). Some enriched species were also increased in bleached coral, including *Lachnospirales* and *Enterobacterales* in *A*. *digitifera*, *Rhodobacterales*, *Rhizobiales*, and *Peptostreptococcales-Tissierellales* in *G*. *fascicularis*, as well as *Rhodobacterales*, *Rhizobiales*, and *Flavobacteriales* in *P*. *pukoensis.* These were upregulated 3.5 to 19.8-fold. Moreover, at the genera level prokaryotic microbiome composition was further observed to be specific in each non-bleached and bleached coral species (Fig. 2D). Analyzing the relative abundance of genus bar plots, which demonstrated a common trend, the proportion of the most dominant genus (such as norank_f_Mitochondria, norank_f_norank_o_Chloroplast, *Prosthecochloris*) in bleached coral decreased compared to non-bleached corals.

**FIG 3:**
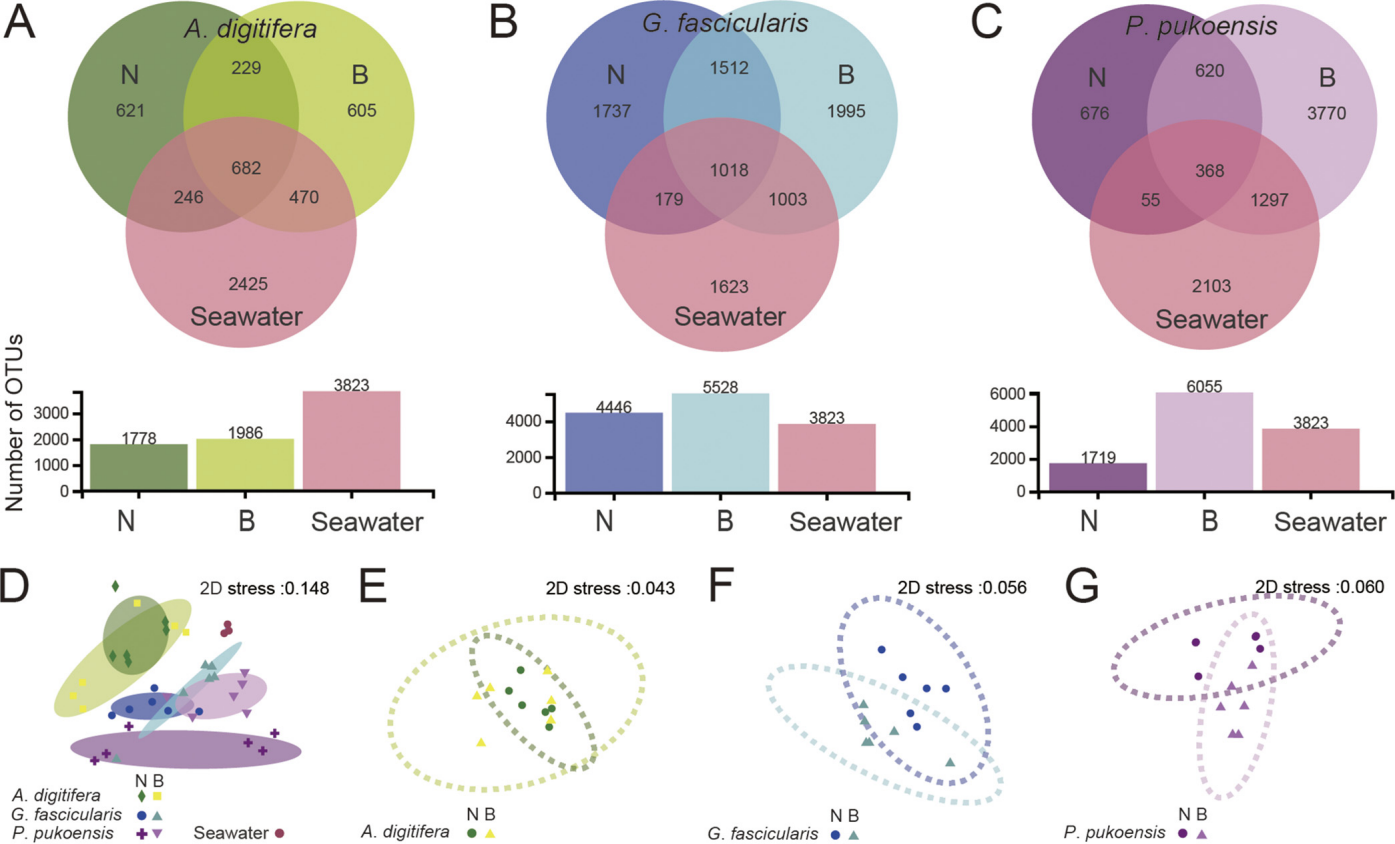
(A to C) Venn diagram for the common and unique number of OTUs among non-bleached and bleached corals and seawater among *A*. *digitifera* (A), *G*. *fascicularis* (B), and *P*. *pukoensis* (C). Prokaryotic community structure and relative dispersion of the non-bleached and bleached coral species and seawater samples, analyzed with nonmetric multidimensional scaling (NMDS) plots using Bray-Curtis dissimilarity. (D) Three coral species (non-bleached and bleached) and seawater. (E to G) *A*. *digitifera*, *G*. *fascicularis*, *and P*. *pukoensis* in non-bleached and bleached model, respectively. N, non-bleached; B, bleached.

Unique and shared OTUs at different conditions in each coral species and seawater are shown in the Venn diagrams in Fig. 3. OTU numbers found uniquely in non-bleached and bleached *P*. *pukoensis* were 676 OTUs and 3,770 OTUs (Fig. 3C), respectively. The number of shared OTUs between was markedly higher in bleached corals and seawater than in non-bleached corals among the three corals. Based on this, we speculated that the symbiotic bacteria of bleached coral were more likely to be affected by the surrounding seawater, showing stronger convergence. In contrast, the healthy corals were more capable of shaping their own bacterial community composition, showing stronger heterogeneity.

Bacterial beta diversity was quantified via nonmetric multidimensional scaling analysis (NMDS) analysis based on Bray-Cutis dissimilarities, which further revealed the community separation among coral and seawater OTUs. The bacterial communities of coral formed distinct clusters with significant differences being found at the OTU level (ANOMIS test) (Fig. 3D). The bleached *G*. *fascicularis* (Fig. 3F; stress: 0.056; *P* value = 0.023) and *P*. *pukoensis* (Fig. 3G; stress: 0.060; *P* value = 0.015) bacterial communities demonstrated a significant separation from their healthy communities, apart from the *A*. *digitifera* samples in Fig. 3E (stress: 0.043; *P* value = 0.353). The bacteria structure of *G*. *fascicularis* and *P*. *pukoensis* was significantly different in non-bleached and bleached samples. This may be because bleaching facilitates high biodiversity of bacteria due to the dramatic migration from surrounding seawater bacteria. This is one source of opportunistic and potentially pathogenic bacteria that cause coral disease and bleaching. Unlike *G*. *fascicularis* and *P*. *pukoensis*, the difference in the diversity of *A*. *digitifera* was not obvious, indicating that its symbiotic bacteria have a certain buffer or tolerance when facing stress.

### Specific bacteria between corals.

**FIG 4:**
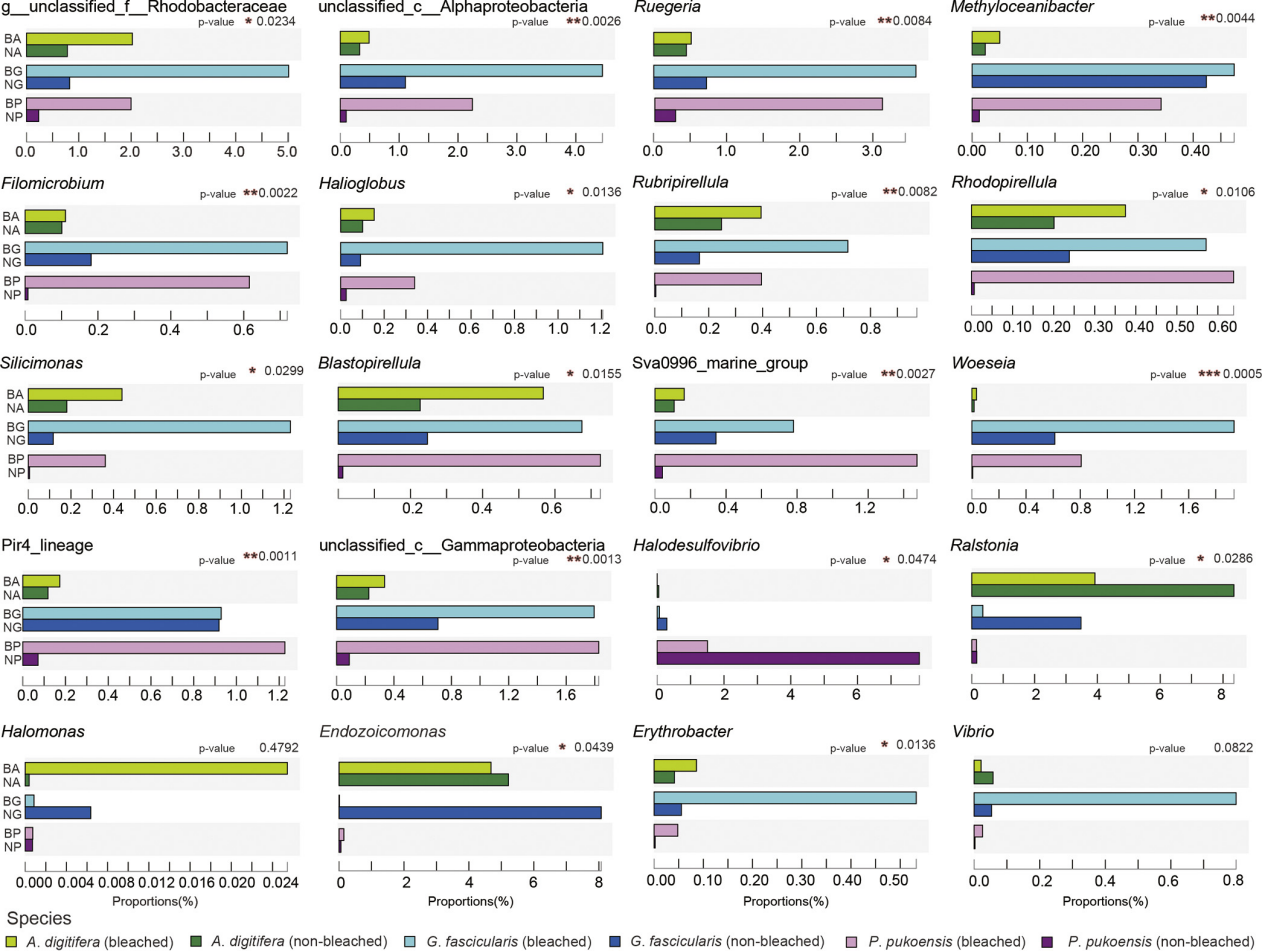
Distribution of the relative abundance of the specific bacteria between corals.

Despite the relatively low bacterial abundance at the genus level, this was considered the primary reason for the bacterial shifts in the phenotypic status of the two corals. As shown in Fig. 4, 14 genera were significantly higher in bleached samples [unclassified_f_Rhodobacteraceae (*P* = 0.023), unclassified_c_Alphaproteobacteria (*P* = 0.002), *Ruegeria* (*P* = 0.008), *Methyloceanibacter* (*P* = 0.004), *Filomicrobium* (*P* = 0.002), *Halioglobus* (*P* = 0.013), *Rubripirellula* (*P* = 0.008), *Rhodopirellula* (*P* = 0.010), *Silicimonas* (*P* = 0.299), *Blastopirellula* (*P* = 0.015), Sva0996 marine group (*P* = 0.002), *Woeseia* (*P* = 0.0005), Pir4 lineage (*P* = 0.001), and unclassified_c_Gammaproteobacteria (*P* = 0.001)] compared with non-bleached. These results indicated that those genera may be associated with bleached coral and bacterial dysbiosis.

The response species also have a strong host correlation. Taxa such as norank_Mitochondria (*P* = 0.0001) and *Halodesulfovibrio* (*P* = 0.047) were particularly enriched in *A*. *digitifera*, and *Prosthecochloris* (*P* = 0.036) was highly enriched in *P*. *pukoensis*. In addition, norank_Chlroplast (*P* = 0.023) and *Ralstonia* (*P* = 0.028) were observed higher in *A*. *digitifera* and *G*. *fascicularis*. The relative abundances of these genera in bleached coral samples were significantly lower than in non-bleached samples, which may suggest their vital function in non-bleached corals. Moreover, the shifts of the individual bacterial genera were dependent not only on their coral species but also on host conditions.

Bacterial profiles also varied from coral species to species. Probiotics and opportunistic and infectious pathogens play an important role in affecting coral health by impacting the functioning of the coral holobiont to suit the prevailing environmental conditions (18, 33). Here, we observed that the relative abundance of some probiotics (*Erythrobacter* and *Ruegeria*) increased in the bleached corals. In comparison, the relative abundance of other probiotics (*Halomonas*, *Endozoicomonas*) and pathogens (*Vibrio*) varied by sample.

### Interactions between non-bleached and bleached coral bacteria.

**FIG 5:**
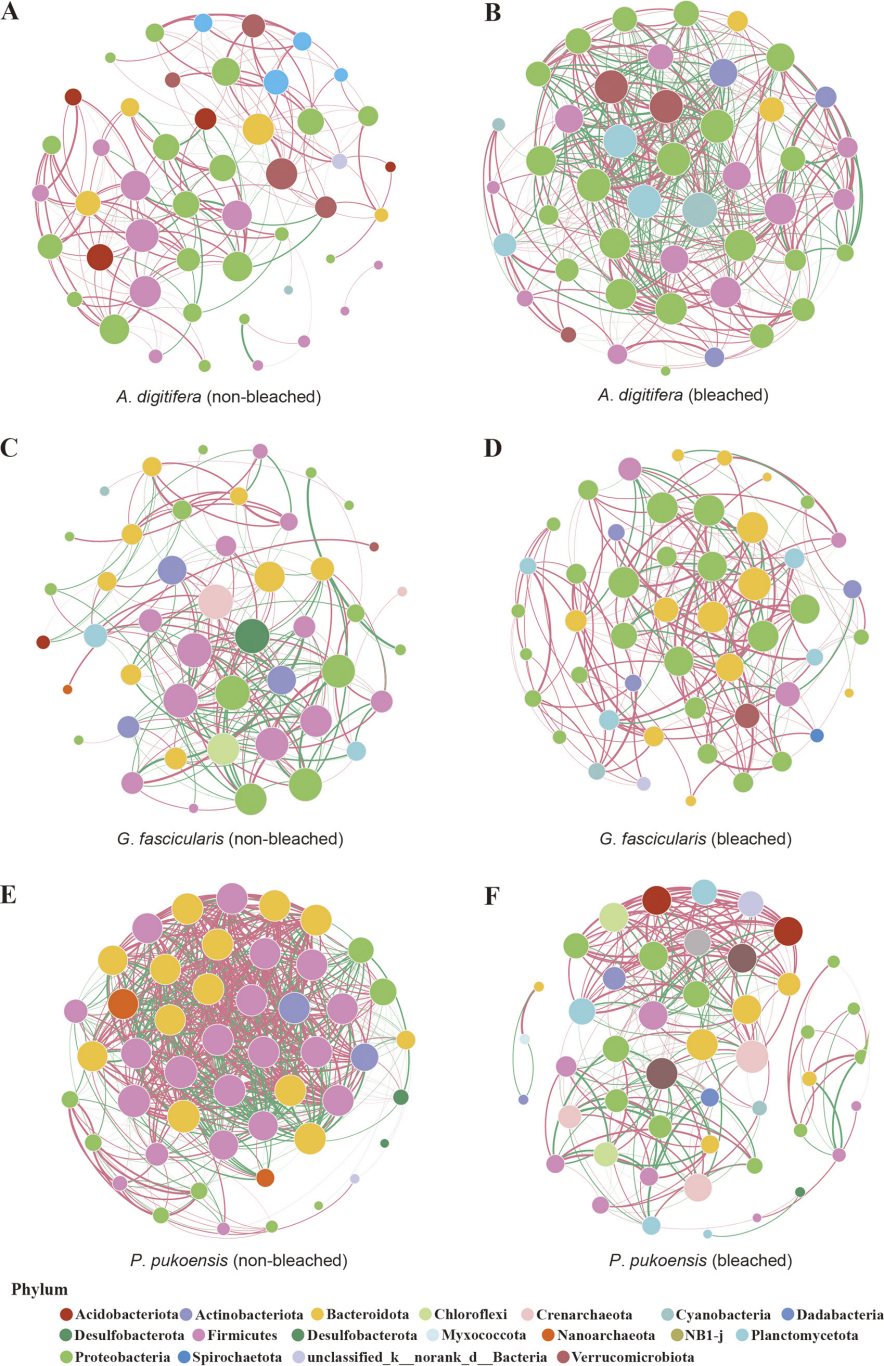
Co-occurrence network of coral bacterial communities at the genus level between two conditions. (A, C, E) Non-bleached. (B, D, F) Bleached. The size of the nodes is proportional to the average relative abundance of the genera. Edge thickness linking two nodes is proportional to the absolute value of the Spearman’s correlation coefficients. Node colors are used to differentiate between phyla. Green edges indicate the co-occurrence of connected nodes, pink edges denote mutual exclusivity, and the width of edges reflects the strength of interaction.

To investigate how interactions with bacterial genera and the complex patterns of interrelationships shifted with bleaching, the topological properties of networks were calculated to reconstruct a genus co-occurrence network in each coral species. The bacteria from each non-bleached and bleached coral species and their individual networks are presented in Fig. 5. In the resulting networks, each node was a bacterial genus with green edges indicating a positive connection (co-occurrent) and pink edges denoting a negative connection (mutual exclusion). The network from bleached *A*. *digitifera* (45 nodes and 444 edges) was more complex than non-bleached (48 nodes and 189 edges). In contrast, the bacterial network of the bleached *P*. *pukoensis* group (48 nodes and 632 edges) had more edges than the non-bleached group (24 nodes and 258 edges). There was no change between non-bleached (46 nodes and 217 edges) and bleached *G*. *fascicularis* (49 nodes and 202 edges).

Networks for the six groups were constructed with a frequency of >50%. According to the calculated properties, the coral-associated bacteria had higher positive edge correlations among all non-bleached and bleached samples. Stable coexistence in a community represents high modularity (34). We found that community networks decreased in modularity as the negative connection increased (Table S3). The ratio of negative edges in the non-bleached *A*. *digitifera* network was approximately 1/3 (positive edges = 149; negative edges = 40) with a modularity of 0.435, whereas the ratio of negative edges in the bleached group was 1/2 (positive edges = 233; negative edges = 211) with a modularity of 0.228. The ratio of negative connections of *G*. *fascicularis* and *P*. *pukoensis* were both 1/3 in the bleached groups with a higher modularity (modularity index was 0.491 and 0.410, respectively) compared to the non-bleached groups (the ratio of negative edges were both 1/2 and modularity index was 0.302 and 0.102, respectively), indicating that bacterial communities with high modularity exist with more positive connections, which are dominant in more stable networks, rather than in negative associations between taxa.

In bleached samples, *Proteobacteria* was the dominant phylum with the most connections with other phylum and was increasingly common compared to non-bleached coral networks, especially in *A*. *digitifera* (Fig. 5A and B) and *G*. *fascicularis* (Fig. 5C and D) groups. In non-bleached *P*. *pukoensis* (Fig. 5E), *Firmicutes*, and *Bacteroidota* were the most dominant hubs with a high level of centrality degrees, abundance of these two hubs prevented the bleached *P*. *pukoensis* group (Fig. 5F) from forming a modularized community network (modularity index = 0.410; Table S3). Similarly, compared with non-bleached *G*. *fascicularis*, the modularity index of 0.491 in the bleached group, suggested that the network had a modular structure. Conversely, the lowest network density of 0.168 was observed in non-bleached *A*. *digitifera*, which had a higher modularity index (modularity index = 0.435; Table S3) than the bleached group. The co-occurrence network showed that bacteria in different corals were significantly different depending on host species and status, they were more modular, and they were dominated by positive co-occurrences.

### Functional profiling of bacterial communities in corals.

To study the functional alternative of bacterial communities in the non-bleached and bleached coral species, the PICRUSt2 bioinformatics tool was used to predict the functional potential and profiles of the coral-associated bacteria based on 16S amplicon sequencing profiles. A level 3 of Kyoto Encyclopedia of Gene and Genomes (KEGG) categories was obtained between the coral samples as shown in Fig. S1. Different patterns in the frequencies of these categories by two conditions of coral species were identified, exhibiting a high frequency of metabolic pathways, biosynthesis of secondary metabolites, microbial metabolism in diverse environments, amino acid biosynthesis, carbon metabolism, and nucleotide metabolism (pyrimidine metabolism). However, other categories such as energy metabolism (methane metabolism, carbon fixation pathway in prokaryotes, and sulfur metabolism), amino acid metabolism (arginine and proline metabolism and arginine biosynthesis lysine degradation), metabolism of cofactors and vitamins (pantothenate and CoA biosynthesis, nicotinate, and nicotinamide metabolism) were less frequent in all groups. *G*. *fascicularis* and *P*. *pukoensis* had a more similar trend involved in the relative frequencies of most categories and were increased in the bleached groups except for *A*. *digitifera* samples.

### Linkage of abiotic and biotic to coral health status.

**FIG 6:**
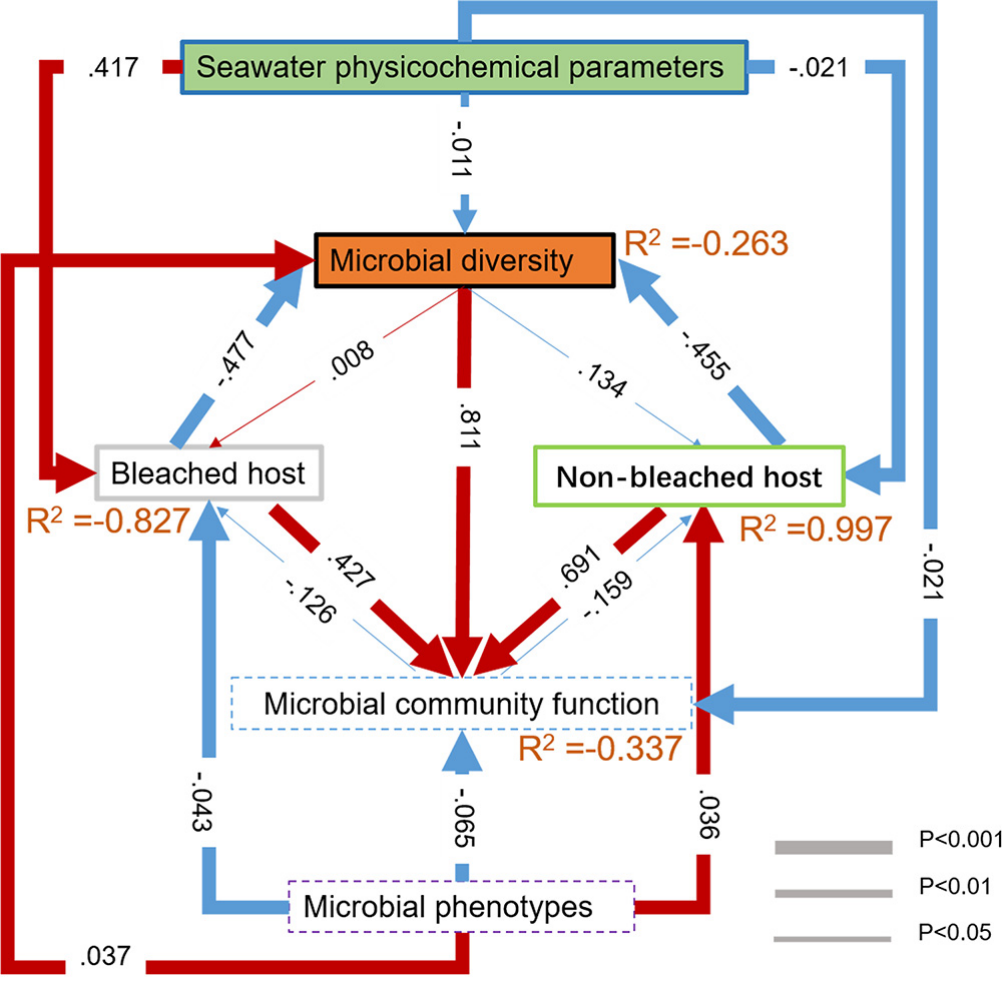
Structure equation modeling showing the relative influence between seawater abiotic and biotic coral associated with microbial factors on coral status. Response variables are represented as solid and predictor variables are represented as bold-dashed boxes. Red and blue arrows indicate positive and negative relationships, respectively. Proportion of variance explained (R^2^) is represented by orange numbers for each response variable. Significant paths imply a causal influence of different variables with arrow widths proportional to the degree of influence, as shown in the bottom right. Arrow numbers represent the standardized path strength.

Structural equation modeling (SEM) was performed to test the relationships or directional influences among coral, microbiome, and environment (Fig. 6). Our results showed that coral status was directly influenced by seawater physicochemical parameters (salinity, temperature, Fe^3+^, silicate, ammonium, nitrite, nitrate, phosphate, pH, and ChI *a*) (*P* < 0.001) and the predictor microbial phenotypes (*P* < 0.001) (Table S4). In addition, the host had a strong direct negative effect on microbial diversity (*P* < 0.001) and a strong direct positive effect on microbial community function (*P* < 0.001), which induced several effects (Fig. 6; Table S5). These results suggested that the host was the dominant determinant of the progress of microbial diversity and function.

## DISCUSSION

Coral ecosystems are facing a dramatic increase in the frequency and intensity of stressors (35), such as heat waves (36, 37), EI Nino-Southern oscillation, and La Nina events (28, 38–42). Moreover, outbreaks of crown-of-thorns starfish, typhoons, diseases, and strong solar radiation also can cause coral bleaching (43, 44). The ways in which coral holobionts respond to bleaching events are often complex and spatiotemporally heterogeneous because of different stress tolerances of coral species, namely, induced coral acclimation and extrinsic environmental factors.

In this study, the bacterial communities associated with three bleached and non-bleached coral species, *A. digitifera*, *G. fascicularis*, and *P. pukoensis*, were characterized. From the overall results, we found that different coral species possessed distinct bacterial communities through NMDS plots and composition analysis, suggesting that coral taxonomy is the major determinant for the symbiotic bacterial structure and composition variations. As shown previously, the top bacterial phyla in all the coral samples were *Proteobacteria*, *Cyanobacteria*, *Actinobacteriota*, and *Firmicutes* (44). Despite relatively stable coral-bacteria associations at the phylum level, the composition of the three target coral species had different diversities and relative abundance when analyzed at lower taxonomic levels (such as class and order levels), further suggesting that coral bacteria are diverse and host specific.

There are three main reasons for the host dependence of coral symbiotic bacteria. First, sensitivity to environmental pressures induces bleaching. Different coral species displayed variant degrees of bleaching and disease susceptibility as reported in several studies (45–47). There is mounting evidence to suggest that *Porites* and *Galaxea* were relatively resistant to heat tolerance and undergo lower mortality and bleaching rates compared to *Acropora* (40, 48, 49). In this study, we observed a higher number of OTUs in *P*. *pukoensis* and *G*. *fascicularis* compared to *A*. *digitifera*, suggesting that *P*. *pukoensis* and *G*. *fascicularis* exhibit higher bleaching thresholds. The host symbiont can obtain or lose high-species variability, depending on the species’ own capacity to resist stressor intensity, symbiotic partnership, and energy metabolism level (48, 49). Differences in the bacteria community structure of coral species are also related to the host’s physiological characteristics of the test species. Among the three corals, *A*. *digitifera* and *G*. *fascicularis* have a larger specific surface area, whereas *P*. *pukoensis* has a relatively smaller specific surface area. Therefore, to some extent, the bacteria of *P. pukoensis* had different characteristics than the other two coral species in this study.

The second reason may be lifestyle. Fast-growing branching coral taxa, such as *Acropora*, are normally highly susceptible to thermal stress (50, 51). The contrasting response of bleaching susceptibility also has been observed in other studies (50, 52, 53). Taking *Acropora* as an example, its individual life history traits are possible factors shaping its rapid adaptation potential (50). Most autotrophic coral holobionts with low heterotrophic capacity (*A. digitifera*) reflect a specialized strategy at low microbiome flexibility for microbiome adaptation to environmental change. Accordingly, *A. digitifera* harbors a highly flexible microbiome, which is thought to contribute to coral holobiont plasticity and adaptation.

The third possible reason is the ecological adaptation strategy. Changes in bacterial community structure tend to show the potential mechanism with metabolic flexibility and functional redundancy (54). The fluctuations of *A. digitifera* and *P. pukoensis* bacteria composition influence the host health outcome. These findings suggest that coral species exhibit different degrees of flexibility in holobiont structure and composition.

Normal coral bacteria play an important role in maintaining the host health. Thus, it is important to understand the dynamics of the coral-associated bacterial community structures in healthy and bleached corals. An increase in bacterial diversity often accompanies the holobiont response to stress as a result of emerging opportunistic taxa that are otherwise absent or suppressed (55). Here, the order of alpha diversity in bleached samples was *G*. *fascicularis*, *P*. *pukoensis*, and *A*. *digitifera*. Venn diagrams showed that the majority of OTUs were recorded in bleached groups, which have a large percentage of OTUs shared with environmental samples (seawater) compared to non-bleached groups. A possible reason may be that the symbiont provides an entry niche for opportunistic taxa from seawater that can be able to colonize bleached coral hosts during bleaching events. Non-bleached coral communities can escape thermal bleaching in the same biotope and might benefit from their stable immune systems (56). Under this situation, alpha diversity was increased in the bleached samples. However, our results differ from those obtained by Pollock et al. (57) who conclude that bleached corals had half the number of healthy coral OTUs. This may be because coral immune capacity may be suppressed in bleached coral holobionts with specific microorganism recruitment from the environment, which affects a coral’s susceptibility to disease (57).

*Rhodobacteraceae*, *Flavobacteriaceae*, and *Synechococcaeae* were significantly increased in bleached coral samples and were the dominant taxa in seawater. Based on their thermal sensitivity, these genera have the potential as biomarkers of high temperature in coral ecosystems (58). Meanwhile, shifts in coral-associated bacterial communities may contribute more to the resilience and survival of coral holobionts to environmental disturbances (59, 60). The beneficial or probiotic bacteria of corals, such as *Erythrobacter*, *Endozoicomonas*, and *Ruegeria*, occupied a relatively higher proportion in bleached coral and seem to be a defensive mechanism, whereas pathogens (for example, *Vibrio*) were significantly increased in the bleached *G*. *fascicularis* group (61). In contrast, the relative abundance of *Vibrio* in *A*. *digitifera* and *P*. *pukoensis* had lower abundance and there was no significant difference between the bleached and non-bleached corals. One possible explanation could be that *A*. *digitifera* and *P*. *pukoensis* have stronger resistance/resilience than *G*. *fascicularis*, which reduced colonization by the opportunistic *Vibrio* from surrounding water or sediments. Previous reports have also shown that a lower abundance of *Vibrio* was found in bleached *Diploria strigosa* and *Siderastrea sidereal*, both Caribbean coral species, while a higher abundance was observed in bleached *Pavona duerdeni* and *Porites lutea* of the Pacific coral reef (62). Similarly, consistent with our study, Krishnaswamy et al. (63) did not observe significant pathogens like *Vibrio* in the diseased *Porites lutea* and suggested that “unique species owning a unique microbial composition” contribute to it (63). Changes in the abundance of *Vibrio* were shown to vary across both coral species and geographic locations. In contrast, *Endozoicomonas* remained stable even under the conditions of coral bleaching and nonbleaching proportionally among *A. digitifera* and *P*. *pukoensis*, except for *G*. *fascicularis*. Most studies reported that the relative abundance of *Endozoicomonas* was reduced with stress, disease, and bleached corals (64). As Ritchie (65) initially discovered, potential pathogens are inhibited by antibiotics secreted by beneficial bacteria associated with corals. Bacterial consortia in corals were manipulated with the increasing of probiotic bacteria, which can help to partially mitigate coral bleaching and alleviate pathogens (32) and benefit coral heat resistance (66).

The topological properties of networks have been increasingly used to define potential bacterial interconnections and community stability (67, 68). Co-occurrence analyses show that non-bleached and bleached corals had a higher proportion of positive than negative correlations. Among the bacterial community networks, except the *A. digitifera*, both bleached *P*. *pukoensis* and bleached *G*. *fascicularis* groups had evident modular architecture, indicating that the bacterial groups showed a reduced influence to pressure. The higher modularity in healthy corals suggests that their bacteria may have more stable synergistic interactions that allow them to respond to external perturbations more efficiently (69). However, the relatively low complexity of the network is regarded as remarkably vulnerable to environmental interference (70). *Proteobacteria*, *Bacteroidetes*, and *Firmicutes* are the most prevalent taxa among coral associated with diverse bacterial assemblages (71), which were shown to have a strong correlation with other taxa in this study. Among these three taxa, the hub *Proteobacteria* were increased in the bleached group, suggesting that they play an important role in constructed microbiota interaction networks when coral holobionts become bleached. Taken together, the complexity of the co-occurrence networks of bacteria in different status was significantly different, indicating that the efficiency of the co-occurrence network hinge on host-holobiont-specific responses to disturbances.

To study the potential roles of microbiota in non-bleached and bleached coral groups, we performed PICRUSt pathway analysis. The microbiota was more involved in basic metabolic pathways, including biosynthesis of secondary metabolites, microbial metabolism in diverse environments, biosynthesis of amino acids, and carbon metabolism. KEGG analysis results showed that bacterial consortia between non-bleached and bleached corals had no obvious differences. Similar results have shown that bacterial metabiotic functions remain conserved after suffering from bleaching events (72, 73). However, opposite results have been found in coral-associated microbiomes that undergo eutrophication stress (74). Structural changes and functional varieties may have different sensitivities to stress. In fact, Krishnaswamy et al. (63) and Pogoreutz et al. (75) speculated that the functional redundancy of coral microorganisms contributes to the limited differences between the healthy and diseased groups. Based on this, we propose that the changed microbial communities associated with the relatively stable functions of coral are explained by the decoupling effect of taxonomy and function (76). This suggests a possible evolutionary strategy that allows bacteria to undergo microevolution to adapt to stressors in their specific host, maintaining stable balance and well-being for the bacterial communities, which might provide stable situations and opportunities for bleached coral recovery. The potential association between bacteria-environment-coral was established by using the SEM analysis. Coral host and seawater physicochemical parameters had a relatively significant impact on bacterial profiles (community and function). Bacterial phenotypes drive a negative influence on bleached corals and a positive influence on non-bleached corals.

### Conclusion.

The current study characterized the bacterial composition of three coral species under non-bleached and bleached conditions. The different coral species maintained relatively conserved bacterial populations, indicating that coral symbiotic bacteria have certain host specificity. However, the community structure for a given host can vary by health status. Higher alpha biodiversity was observed in bleached coral samples, and some indicator bacteria acted as potential biomarkers in bleached individuals. Additionally, the bleached and non-bleached groups had significantly different network modularity profiles. After multiparameter coupling analysis by SEM, the results (Fig. 6) revealed that the coral host has the main effect on microbial diversity and function. This study improves our understanding of the bacterial composition of different coral species exhibiting host dependence and how these symbionts contribute to the coral holobiont’s environmental adaptability. However, it should be noted that the current research is only based on limited samples taken at one time point. In the future, we need to conduct tracking and collect a larger number of sequential samples in the natural environment. In addition, the metabolic potential of this work is based on functional prediction. Metagenomic analysis is needed to clarify the exact functional mechanism of coral symbiotic bacteria.

## MATERIALS AND METHODS

### Sampling and processing.

**FIG 7:**
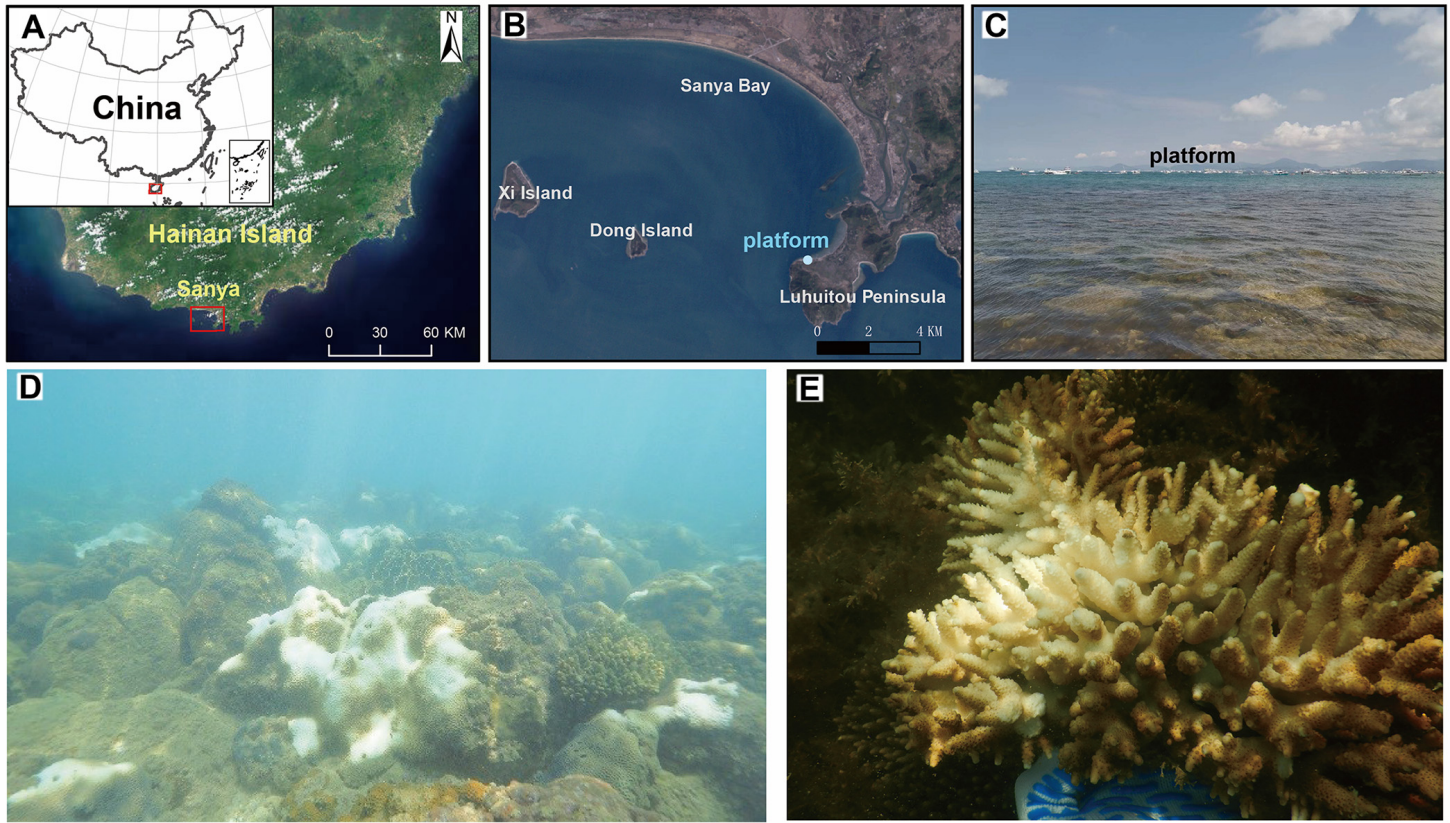
(A) Map of the South China Sea study site. (B) Corals and seawater were sampled at one coral reef site off Luhuitou peninsula coastal, which is marked with a circle. (C) The site is located approximately 200 m northeast of Luhuitou (image of sampling site). (D) *In situ* monitoring of coral bleaching through fixed undersea observation equipment at the Hainan Island. (E) Representative real-time captured image of bleached corallite.

Three types of corals (*Acropora digitifera*, *Galaxea fascicularis*, and *Porites pukoensis*) fragments (approximately 5 to 15 cm each) and seawater were collected on April 4, 2021, in the South China Sea off the coast of Luhuitou, Sanya, Hainan (18°12’N, 109°28’E) (Fig. 7), when the seawater temperature was 24.49 ± 0.29°C at salinity of 32.2 ± 0.2‰, pH of 8.16 ± 0.03. The three coral species were identified based on their characteristic morphological features via an online website http://www.coralsoftheworld.org/page/home/. From this, fragments were collected from each colony of both visually non-bleached and bleached tissue using sterilized scissors and forceps, separately into 100-mL sterile plastic bottles. Surrounding seawater was filtered (3 L) onto 0.2-μm MCE membrane filters (47-mm diameter; Millipore, Billerica, USA) in duplicate. Each filter (*n* = 3) was placed into aseptic centrifugal tubes (50 mL) with a screw cap. All samples were immediately frozen in a solid carbon dioxide and were transported to the laboratory, at which point they were store at −80°C until required.

### DNA isolation and sequencing of microbial symbionts.

Per coral samples (6 × non-bleached *A*. *digitifera*, 6 × bleached *A*. *digitifera*, 6 × non-bleached *G*. *fascicularis*, 6 × bleached *G*. *fascicularis*, 6 × non-bleached *P*. *pukoensis*, and 6 × bleached *P*. *pukoensis*), a fragment of 1 g was weighed and was immediately ground to a fine powder using liquid nitrogen and a mortar and pestle. DNA was extracted from 36 coral samples and 3 surrounding seawater samples using the Dneasy PowerSoil Pro kit (Qiagen, Hilden, Germany) according to manufacturer’s protocols. DNA integrity was verified through 1% (vol/vol) agarose gel electrophoresis, DNA concentration was examined by using Qubit 2.0. DNA purity was measured using Nanodrop (optical density at 260 nm/optical density at 280 nm ratio) and stored at −20°C before use.

In total 39 DNA samples were sent to Majorbio Bio-Parm Technology Co., Ltd. (Shanghai, China) on solid carbon dioxide for next-generation shotgun sequencing. The V4 hypervariable region of bacterial and archaeal 16S rRNA gene was amplified with primers 515FomdF (5′-GTGYCAGCMGCCGCGGTAA-3′) and 806RmodR (5′-GGACTACNVGGGTWTCTAAT-3′) (77, 78). PCR amplification profile was performed using the following program: initial denaturation of 15 min at 94°C, 27 cycles of denaturation at 95°C for 40 s, annealing at 55°C for 40 s, and amplification at 72°C for 40 s, a final elongation step of 10 min at 72°C. All 16S PCR products were purified by E.Z.N.A. Gel Extraction kit (Omega, USA) and sent for sequencing (Majorbio Bio-Pharm Technology Co., Ltd., Shanghai, China) on the Illumina Hiseq2500 platform.

### Read processing, assembly, and data analysis.

All the obtained raw reads were merged, trimmed, quality filtered, and clustered into OTUs at the ≥97% similarity level using Uparse pipeline (79) (version 7.0.1090), which were aligned and compared with the SILVA database v138 with a bootstrap confidence cutoff of 70%. To reduce the nonsystematic variation from amplification bias and sequencing errors, the sequences were rarefied to normalization to the minimum number of reads per sample before calculating the respective alpha diversity (Shannon indices) and richness (Chao1 indices) by Student’s *t* test; the Good’s coverage was calculated by mothur (80) (version 1.30.2) to evaluate OTU similarity level (97%). Venn diagram and bar plot analysis were conducted using the “vegan” package in R (version 3.3.1) for Linux to visualize shifts in the percentage of community abundances on different levels among different samples. NMDS using based on the Bray-Curtis dissimilarity index of the beta diversity of bacterial community among coral and seawater samples was potted in QIIME (version 1.9.1) software.

### Network feature, functional profile, and statistical analysis.

To describe the topological properties of the co-occurrence patterns among microbiome between non-bleached and bleached coral samples, the opened source platform Gephi (version 0.9.2) was used to visualize entity relationship with the network. We selected bacterial genera with a frequency of ≥50% with the Spearman coefficients absolute values ≥0.5 and false discovery rate corrected *P* value of ≤0.05. Each node represents one genus, and its color indicates one phylum. Edges make significant correlations (negative and positive relationships) among nodes. The clustering coefficient, closeness values, modularity, and eigenvectors were calculated to identify the topological properties of six different co-occurrence networks.

Analysis of variance and Tukey’s honestly significant difference *post hoc* analyses identified differentially expressed probiotic microbes between non-bleached and bleached coral samples. The relative abundance of bacterial genera among the six groups (coral samples) was statistically analyzed using the Kruskal-Wallis H test followed by the Scheffé’s *post hoc* test.

To assess the potential functional profiles of the microbiome between the non-bleached and bleached coral samples, PICRUSt2 was used to predict metagenomes from 16S data in the bacterial microbiome. A heatmap of the imputed relative abundances of KEGG pathways in each coral sample predicted by the KEGG catalog (81). In addition, an SEM was analyzed via the online website https://spssau.com (82) to identify the relationship among seawater physicochemical parameters, microbial diversity, predictor factors (microbial community function and phenotype), and coral status. As the abiotic and biotic drivers drive the coral symbiont’s health, the priori model was set as follows: (1) the host, microbial diversity, and community function are driven by abiotic factors; (2) the biotic factors are driving the host status; and (3) the host drives the predictor factors.

### Data availability.

All sequencing runs and data filtering were conducted at Majorbio, Inc. (Shanghai, China), and sequence data utilized for this project were deposited in the NCBI Short Read Archive database and can be accessed with the BioProject (PRJNA922724).
