# Supplementary material for: Bacterial Communities Vary from Different Scleractinian Coral Species and between Bleached and Non-Bleached Corals
Source: Microbiol Spectr. 2023 May 16;11(3):e04910-22. doi: 10.1128/spectrum.04910-22 (PMC10269541; doi:10.1128/spectrum.04910-22)
Supplement: Supplemental file 1 — Fig. S1 and Tables S1 to S5. Download spectrum.04910-22-s0001.pdf, PDF file, 0.6 MB [file spectrum.04910-22-s0001.pdf]

Supplemental information

Figure S1 Functional analysis of the predicted pathways among the non-bleached and bleached coral species by PICRUSt2. The relative abundances of the top 50 pathways for *A. digitifera*, *G. fascicularis*, *P. pukoensis* groups.

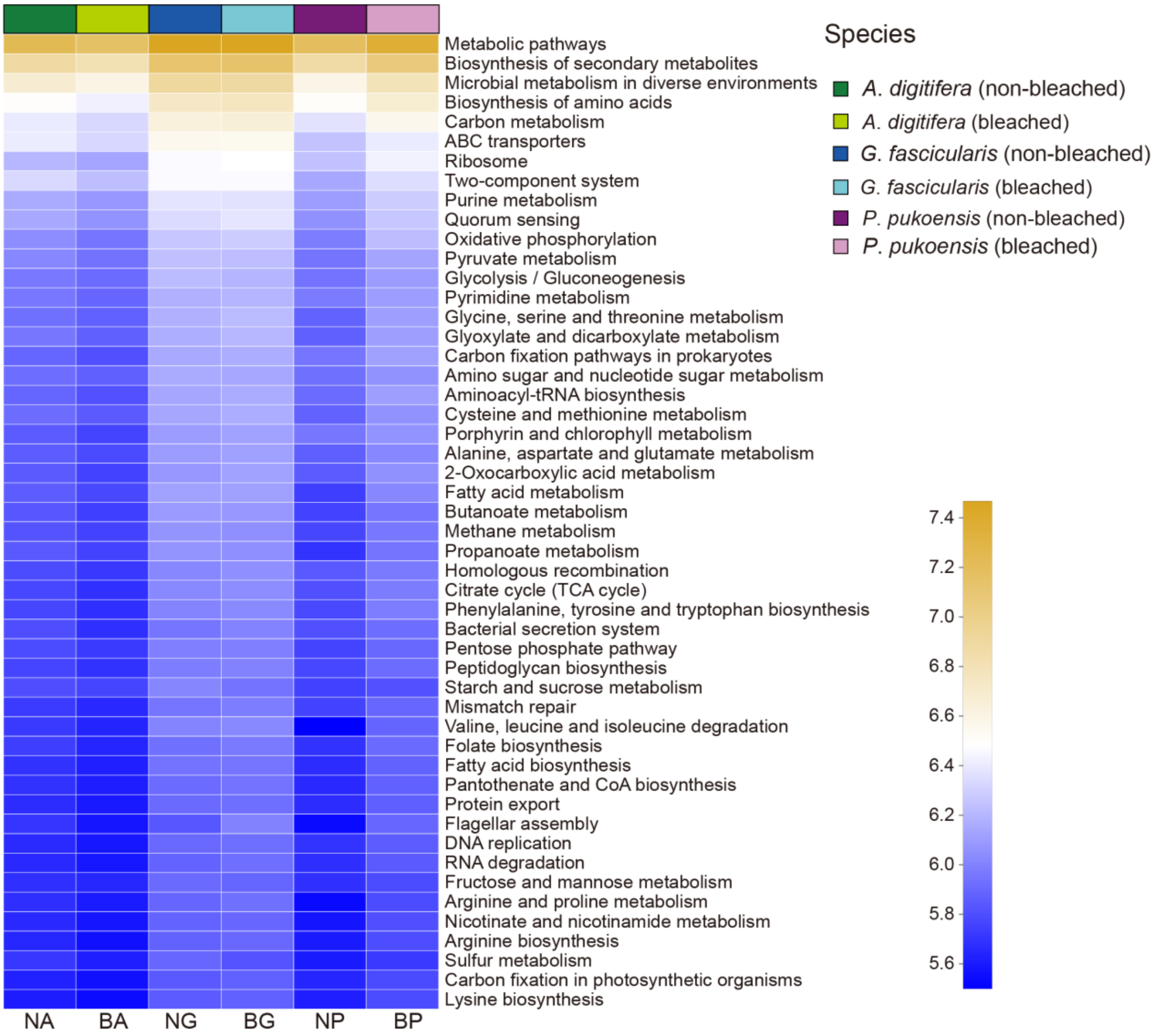

Table S1 Number of sequencing reads, Good's coverage and alpha diversity indices of coral and seawater samples of 16S rDNA profiles, at OTUs level.

**Sample**	**Quality read**	**Shannon**	**Chao 1**	**Good's coverage**	**Average OTUs**
A. digitifera (non-bleached)	431,693	2.4947	580.27	99.44%	459.5
A. digitifera (bleached)	309,922	3.2228	682.07	99.28%	504.17
G. fascicularis (non-bleached)	409,249	4.6951	1524.2	98.49%	1233
G. fascicularis (bleached)	406,583	5.4976	2889.1	96.39%	1920.7
P. pukoensis (non-bleached)	286,803	3.0775	632.07	99.46%	471.83
P. pukoensis (bleached)	377,375	5.3218	2661	96.89%	1873.2
seawater	342,249	5.4158	3989.7	94.97%	2288.7

Table S2 Student's t-test from pairwise comparisons of microbial community profiles.

sample pair	Chao 1 index		Shannon index	
	q-value	***p*-value**	***q-value***	***p*-value**
NAcr vs BAcr	0.8472	0.7655	0.8472	0.3391
NGal vs BGal	0.04254	0.02836	0.04254	0.02514
NPor vs BPor	0.008011	0.004006	0.03884	0.03237
NAcr vs NGal	0.0575	0.03449	0.009913	0.001652
NAcr vs NPor	0.958	0.7695	0.958	0.5342
NGal vs NPor	0.08602	0.02867	0.09321	0.077
BAcr vs BGal	0.002065	0.001228	0.003985	0.003321
BAcr vs BPor	0.01851	0.009257	0.02082	0.01581
BGal vs BPor	0.7437	0.8924	0.8924	0.7252
NAcr vs SW	1.86E-05	6.20E-06	0.004364	0.003636
NGal vs SW	0.005076	0.002276	0.129	0.1075
NPor vs SW	3.08E-07	9.01E-08	0.09947	0.08289
BAcr vs SW	0.000279	0.00014	0.04225	0.0352
BGal vs SW	0.257	0.1133	0.7972	0.7972
BPor vs SW	0.2896	0.1448	0.9416	0.8935

Abbreviations are followed: non-bleached and bleached *Acropora Digitifera*., NAcr and BAcr; non-bleached and bleached *Galaxea Fascicularis*., NGal and BGal; non-bleached and bleached *Porites Pukoensis*., NPor and BPor; seawater., SW.

Table S3 The topological parameters for six co-occurrence networks.

Samples	Nodes	Edges	Positive	Negative	Average nod connectivity	Network diameter	Graph density	Average path length	Modularity index	Average eigenvectors centrality	Average clustering coefficient	Average closeness centrality
A. digitifera (non-bleached)	48	189	149	40	7.875	7	0.168	2.092	0.435	0.130	0.572	0.489
A. digitifera (bleached)	45	444	233	211	19.733	4	0.448	1.593	0.228	0.156	0.714	0.651
G. fascicularis (non-bleached)	46	217	122	105	9.435	5	0.210	1.661	0.302	0.107	0.538	0.561
G. fascicularis (bleached)	49	202	150	52	8.245	8	0.172	2.063	0.491	0.168	0.639	0.542
P. pukoensis (non-bleached)	48	632	410	212	26.332	5	0.560	1.44	0.102	0.183	0.794	0.702
P. pukoensis (bleached)	47	258	145	103	10.979	5	0.239	1.564	0.410	0.128	0.708	0.665

Table S4. The data of relevant environmental parameters.

**Sample group**	**Sample ID**	**Temperature (℃)**	**Salinity (‰)**	**ChI-*a* (μg/L)**	**pH**	**Phosphate (mg/L)**	**Nitrate (mg/L)**	**Nitrite (mg/L)**	**Ammonium (mg/L)**	**Silicate (μM)**
A. digitifera (non-bleached)	LJ_6J	24.60	32.2	15.5	8.17	0.024	0.17	0.012	0.05	14.5
	LJ_1J	24.70	32.3	14.9	8.15	0.022	0.16	0.008	0.045	12.6
	LJ_2J	24.32	32.0	15.3	8.2	0.024	0.16	0.009	0.042	18.6
	LJ_4J	24.55	32.0	14.0	8.15	0.021	0.17	0.014	0.049	14.5
	LJ_5J	24.45	32.4	15.2	8.12	0.022	0.16	0.008	0.05	16.5
	LJ_3J	24.42	32.4	14.9	8.16	0.026	0.16	0.008	0.042	12.1
A. digitifera (bleached)	LJ_1B	24.72	32.1	14.5	8.18	0.026	0.15	0.012	0.052	13.4
	LJ_6B	24.65	32.2	14.6	8.14	0.024	0.16	0.008	0.046	18.3
	LJ_4B	24.44	32.1	15.3	8.15	0.022	0.16	0.012	0.047	14.4
	LJ_2B	24.32	32.0	14.8	8.16	0.021	0.17	0.012	0.052	17.2
	LJ_5B	24.32	32.4	14.7	8.13	0.026	0.16	0.008	0.047	17.2
	LJ_3B	24.44	32.1	15.3	8.19	0.024	0.17	0.011	0.042	13.6
G. fascicularis (non-bleached)	FC_1J	24.81	32.1	15.5	8.15	0.022	0.17	0.012	0.046	13.4
	FC_6J	24.72	32.3	14.8	8.16	0.025	0.16	0.009	0.053	14.4
	FC_5J	24.66	32.2	15.5	8.13	0.026	0.15	0.009	0.05	18.2
	FC_3J	24.32	32.2	15.7	8.2	0.022	0.17	0.011	0.052	14.4
	FC_4J	24.44	32.3	15.3	8.16	0.021	0.16	0.008	0.048	17.2
	FC_2J	24.52	32.2	14.2	8.15	0.024	0.16	0.009	0.046	12.6
G. fascicularis (bleached)	FC_1B	24.32	32.1	15.3	8.13	0.026	0.17	0.012	0.046	18.0

	FC_3B	24.32	32.0	14.6	8.2	0.022	0.16	0.011	0.042	12.8
	FC_4B	24.44	32.0	14.9	8.13	0.025	0.15	0.010	0.048	15.9
	FC_2B	24.58	32.1	15.3	8.15	0.024	0.17	0.012	0.053	17.2
	FC_6B	24.32	32.0	14.8	8.19	0.021	0.16	0.008	0.05	18.2
	FC_5B	24.44	32.2	15.7	8.13	0.022	0.16	0.011	0.05	16.0
P. pukoensis (non-bleached)	BJ_4J	24.26	32.1	15.3	8.2	0.024	0.17	0.012	0.048	17.2
	BJ_5J	24.25	32.1	15.6	8.14	0.024	0.15	0.010	0.05	18.2
	BJ_3J_1	24.72	32.2	14.8	8.16	0.022	0.16	0.009	0.055	16.5
	BJ_3J	24.58	32.0	14.5	8.2	0.025	0.17	0.012	0.042	15.8
	BJ_1J	24.51	32.1	14.8	8.13	0.021	0.16	0.008	0.046	15.8
	BB_1B	24.36	32.1	14.5	8.16	0.022	0.16	0.012	0.053	17.2
P. pukoensis (bleached)	BB_3B	24.68	32.2	15.4	8.15	0.022	0.17	0.012	0.047	16.7
	BB_4B	24.44	32.0	14.8	8.2	0.024	0.17	0.008	0.052	18.2
	BJ_6J	24.57	32.4	15.3	8.15	0.022	0.17	0.008	0.046	16.0
	BB_2B	24.45	32.1	15.4	8.15	0.021	0.17	0.012	0.042	14.4
	BB_5B	24.72	32.3	15.9	8.16	0.022	0.16	0.008	0.047	17.2
	BB_6B	24.42	32.3	15.3	8.15	0.025	0.15	0.010	0.053	17.7
Seawater	W_1	24.12	32.0	14.8	8.13	0.026	0.17	0.012	0.049	18.2
	W_3	24.36	32.4	14.2	8.15	0.021	0.16	0.008	0.05	16.5
	W_2	24.72	32.0	15.3	8.2	0.022	0.16	0.011	0.042	15.2

Table S5. Results of path SEM model: standardized regression coefficients for the entire coral groups.

			SE	CR	*p*-value	Estimate
Microbial diversity	→	Microbial community function	0.099	4.007	0	0.811
Microbial diversity	→	Non-bleached-host	0.028	1.48	0.139	0.134
Microbial diversity	→	Bleached-host	0.049	0.052	0.959	0.008
Microbial diversity	→	Seawater Physicochemical parameters	0.083	2.289	0.022	7.897
Microbial diversity	→	Microbial phenotypes	0.084	-3.403	0.001	-6.833
Microbial community function	→	Microbial diversity	0.238	-0.759	0.448	-0.088
Microbial community function	→	Non-bleached-host	0.085	-1.191	0.234	-0.159
Microbial community function	→	Bleached-host	0.155	-0.517	0.605	-0.126
Microbial community function	→	Seawater Physicochemical parameters	0.126	-2.095	0.036	-5.397
Microbial community function	→	Microbial phenotypes	0.068	-3.359	0.001	-2.683
Non-bleached-host	→	Microbial diversity	0.125	-12.248	0	-0.477
Non-bleached-host	→	Microbial community function	0.027	40.002	0	0.691
Non-bleached-host	→	Bleached-host	0.06	-16.983	0	-1.017
Non-bleached-host	→	Seawater Physicochemical parameters	0.055	-11.119	0	-7.901
Non-bleached-host	→	Microbial phenotypes	0.118	8.539	0	7.496
Bleached-host	→	Microbial diversity	0.125	11.432	0	0.445
Bleached-host	→	Microbial community function	0.027	24.455	0	0.427
Bleached-host	→	Non-bleached-host	0.061	-17.234	0	-1.048
Bleached-host	→	Seawater Physicochemical parameters	0.049	-14.984	0	-9.519
Bleached-host	→	Microbial phenotypes	0.118	-6.273	0	-5.512
Seawater Physicochemical parameters	→	Microbial diversity	0.003	-143.719	0	-0.011
Seawater Physicochemical parameters	→	Microbial community function	0.005	-80.426	0	-0.021
Seawater Physicochemical parameters	→	Non-bleached-host	0.03	-9.104	0	-0.021
Seawater Physicochemical parameters	→	Bleached-host	0.008	683.899	0	0.417
Seawater Physicochemical parameters	→	Microbial phenotypes	0.002	-1329.318	0	-1.242

Microbial phenotypes	→	Microbial diversity	0.009	104.048	0	0.037
Microbial phenotypes	→	Microbial community function	0.012	-65.025	0	-0.065
Microbial phenotypes	→	Non-bleached-host	0.035	7.619	0	0.036
Microbial phenotypes	→	Bleached-host	0.077	-4.123	0	-0.043
Microbial phenotypes	→	Seawater Physicochemical parameters	0.013	423.206	0	9.88
Microbial diversity	→	Simpson	0.013	-10.337	0	-0.891
Microbial diversity	→	Sobs	61.982	6.118	0	0.729
Microbial diversity	→	Chao	93.636	5.703	0	0.702
Microbial diversity	→	Shannon	-	-	-	1
Microbial community function	→	Quorum sensing	0	24368150	0	0
Microbial community function	→	Purine metabolism	0	89350.558	0	0
Microbial community function	→	Two-component system	0	11346763	0	0
Microbial community function	→	Ribosome	0	21463526	0	0
Microbial community function	→	ABC transporters	0	123402.1	0	0
Microbial community function	→	Carbon metabolism	0	162174.88	0	0
Microbial community function	→	Biosynthesis of amino acids	0	766876.1	0	0
Microbial community function	→	Microbial metabolism in diverse environments	0	23776.082	0	0
Microbial community function	→	Biosynthesis of secondary metabolites	0	13910.038	0	0
Microbial community function	→	Metabolic pathways	-	-	-	0
Seawater Physicochemical parameters	→	Fe^{3+}	17.932	-1.012	0.311	-0.219
Seawater Physicochemical parameters	→	Silicate	22.334	-0.602	0.547	-0.109
Seawater Physicochemical parameters	→	Ammonium	13.97	0.394	0.693	0.068
Seawater Physicochemical parameters	→	Nitrite	16.888	-1.429	0.153	-0.531
Seawater Physicochemical parameters	→	Nitrate	3.573	-0.89	0.374	-0.179
Seawater Physicochemical parameters	→	Phosphate	6.785	0.94	0.347	0.194
Seawater Physicochemical parameters	→	pH	0.195	-1.351	0.177	-0.421
Seawater Physicochemical	→	Chl-*a*	19.055	1.089	0.276	0.248

parameters						
Seawater Physicochemical parameters	→	Salinity	23.328	1.419	0.156	0.997
Seawater Physicochemical parameters	→	Temperature	-	-	-	0.25
Microbial phenotypes	→	Stress Tolerant	1.717	1.816	0.069	0.996
Microbial phenotypes	→	Potentially Pathogenic	1.727	1.816	0.069	0.994
Microbial phenotypes	→	Gram Positive	0.499	-0.01	0.992	-0.002
Microbial phenotypes	→	Gram Negative	0.499	0.009	0.993	0.001
Microbial phenotypes	→	Forms Biofilms	0.63	1.138	0.255	0.237
Microbial phenotypes	→	Facultatively Anaerobic	0.373	1.579	0.114	0.47
Microbial phenotypes	→	Contains Mobile Elements	1.563	1.783	0.075	0.841
Microbial phenotypes	→	Anaerobic	1.331	-1.555	0.12	-0.449
Microbial phenotypes	→	Aerobic	-	-	-	0.29
